# Supplementary material for: Sludge Derived Carbon Modified Anode in Microbial Fuel Cell for Performance Improvement and Microbial Community Dynamics
Source: Membranes (Basel). 2022 Jan 20;12(2):120. doi: 10.3390/membranes12020120 (PMC8879649; doi:10.3390/membranes12020120)
Supplement: Supplementary file 1 [file membranes-12-00120-s001.zip › membranes-1534314-supplementary.pdf]

Supporting Information Available

Sludge Derived Carbon Modified Anode in Microbial Fuel Cell for Performance Improvement and Microbial Community Dynamics

Kaili Zhu, Yihu Xu, Xiao Yang, Wencai Fu, Wenhao Dang, Jinxia Yuan and Zhiwei Wang *

Key Laboratory of Clean Pulp & Papermaking and Pollution Control of Guangxi, College of Light Industry and Food Engineering, Guangxi University, Nanning 530004, China; zhukaili26@163.com (K.Z.); xuyihuzwj@163.com (Y.X.); yxiao0112@163.com (X.Y.); fl392597@163.com (W.F.); wenhaodang14@126.com (W.D.); yuan09@syr.edu (J.Y.)

* Correspondence: author: wangzhiwei@gxu.edu.cn

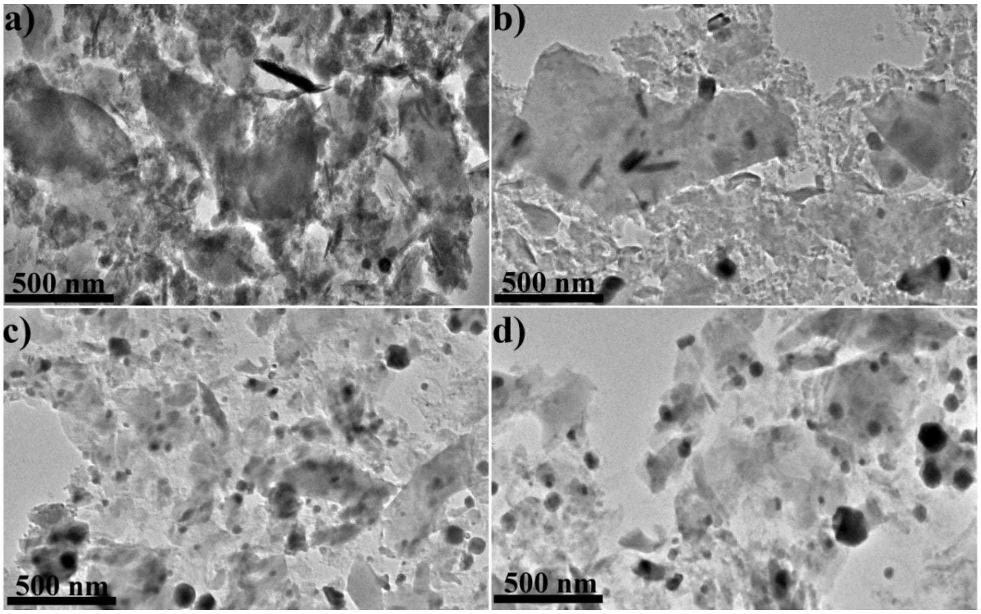

Figure S1. TEM images of SC samples under different carbonization temperature: a) SC600, b) SC800, c) SC1000, and d) SC1200

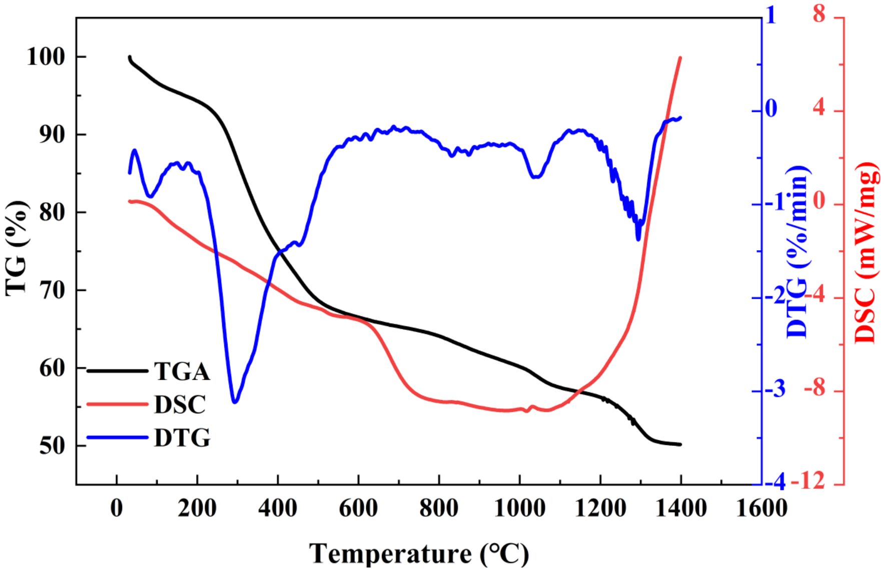

Figure S2. TG-DSC plots of raw sludge under N_2 atmosphere.

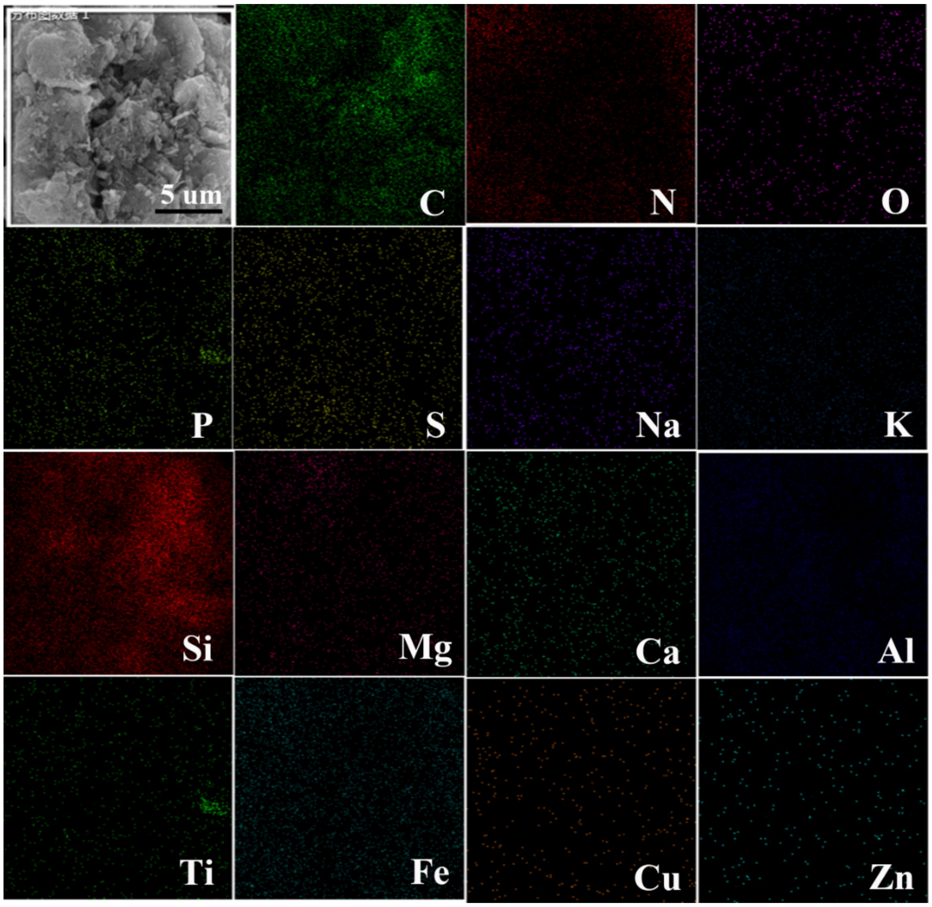

Figure S3. EDX element mappings of the SC1000 sample.

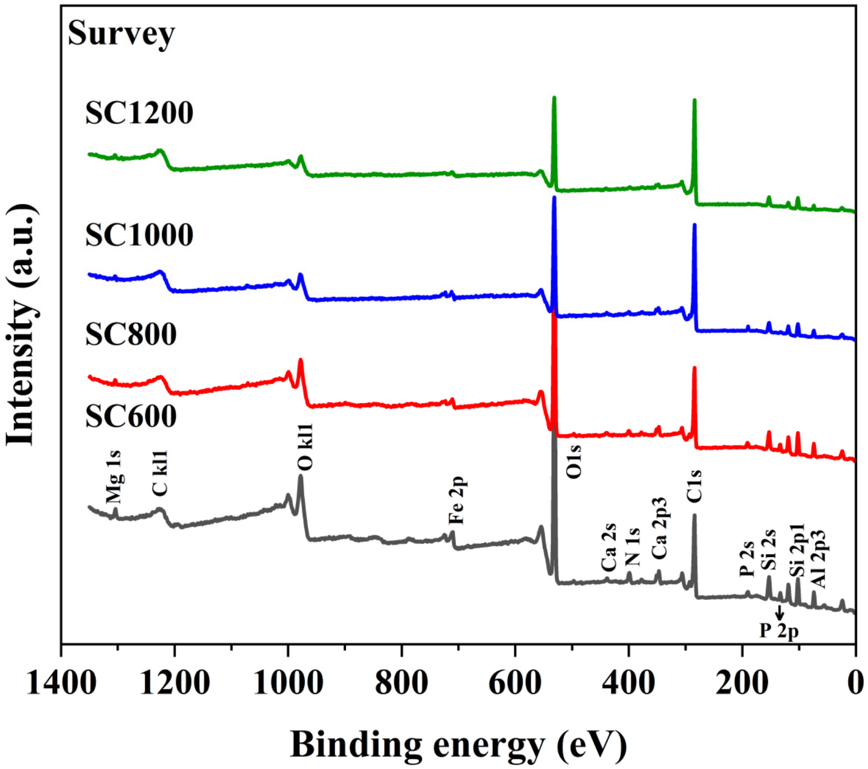

Figure S4. XPS survey spectrum of the SC samples under different carbonization temperature.

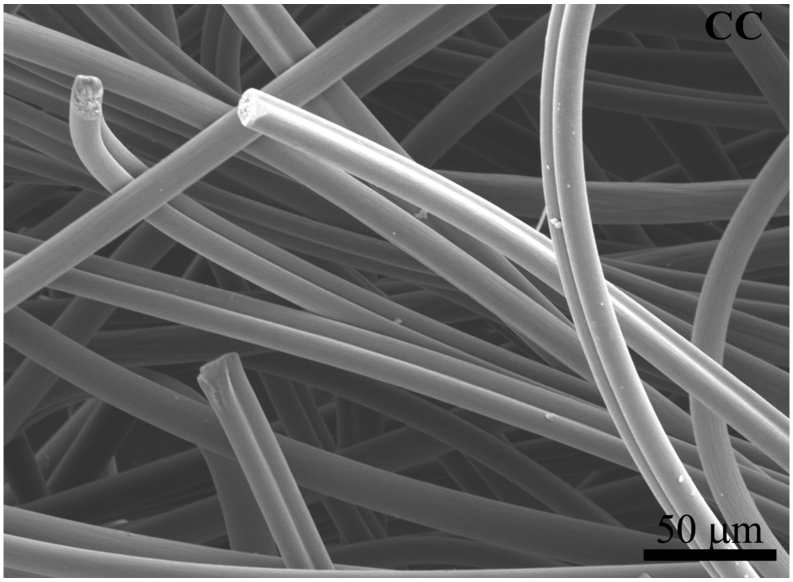

Figure S5. SEM image of bare carbon cloth electrode.

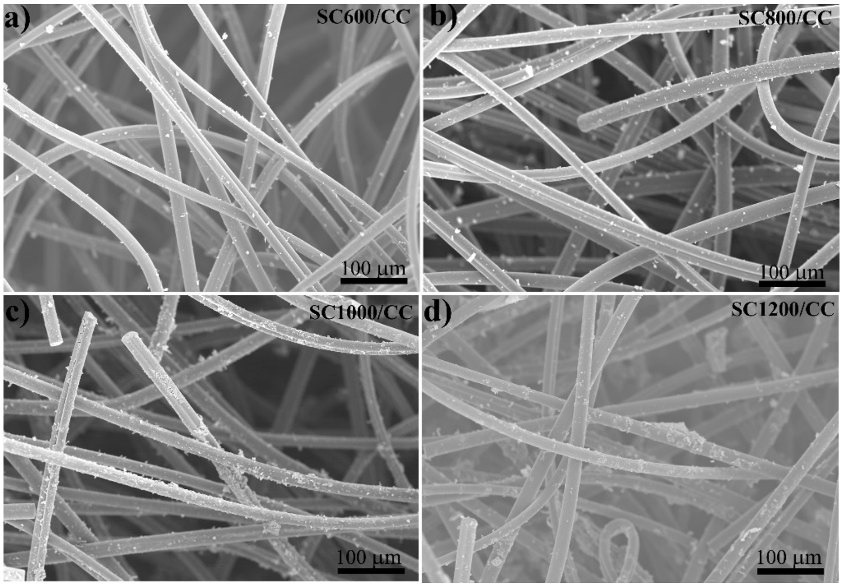

Figure S6. SEM images of loaded electrodes with different SC samples.

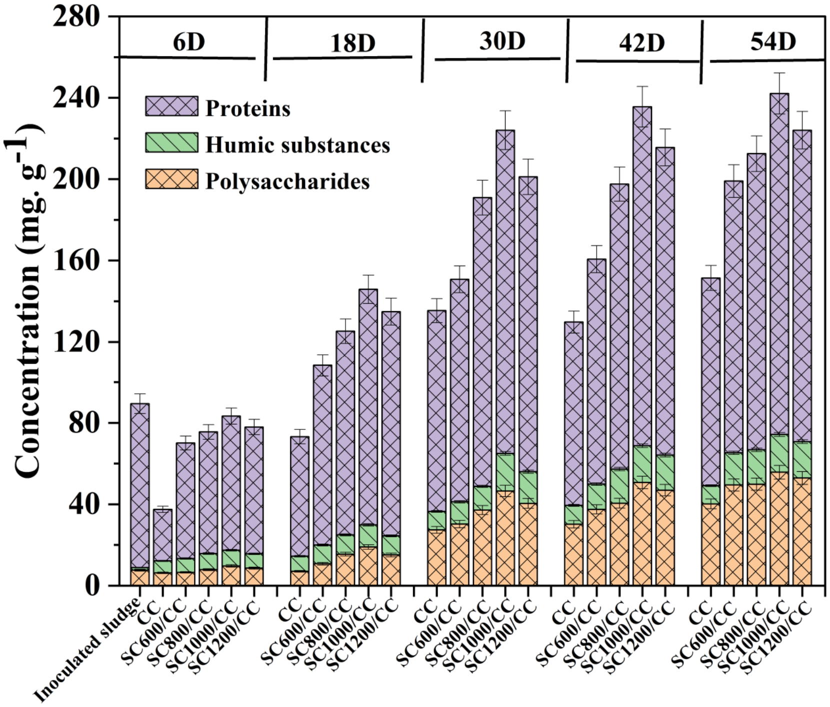

Figure S7. The composition and content of extracellular polymer substances for inoculated sludge and different anode biofilms at MFCs

Studies have shown that EPS plays an important role in promoting microbial metabolism, continuous generation and transfer of electrons, and maintenance of the stability of EABFs [1]. The EPS composition of all anode biofilms was mainly proteins, and the protein content was much higher than that of polysaccharides and humic substances (Figure S7). Interestingly, the change in EPS concentration was positively correlated with changes in protein and polysaccharide concentrations. It has been reported that proteins play an important role in bacterial adhesion, signal molecule transmission, and extracellular electron transfer. For example, cytochrome, as a redox-active component, is conducive to rapid electron transfer [2, 3]. Therefore, the increase in protein concentration in the EPS is presumably related to the expression of cytochrome. Compared to the CC electrode, SC electrodes can induce the regulation of EPS and significantly increase the contents of proteins, polysaccharides, and humic substances in EPS. Polysaccharides can promote adhesion between bacteria and electrodes, and humic substances can promote the degradation of organic pollutants. It has been reported that humic substances can enhance oxidation activity and can be used as a medium to enhance electricity production and catalyze long-distance EET reactions [4, 5, 6]. In particular, the CC anode of SC1000 had the highest EPS concentration, which further confirmed its efficient organic degradation and electrocatalytic ability.

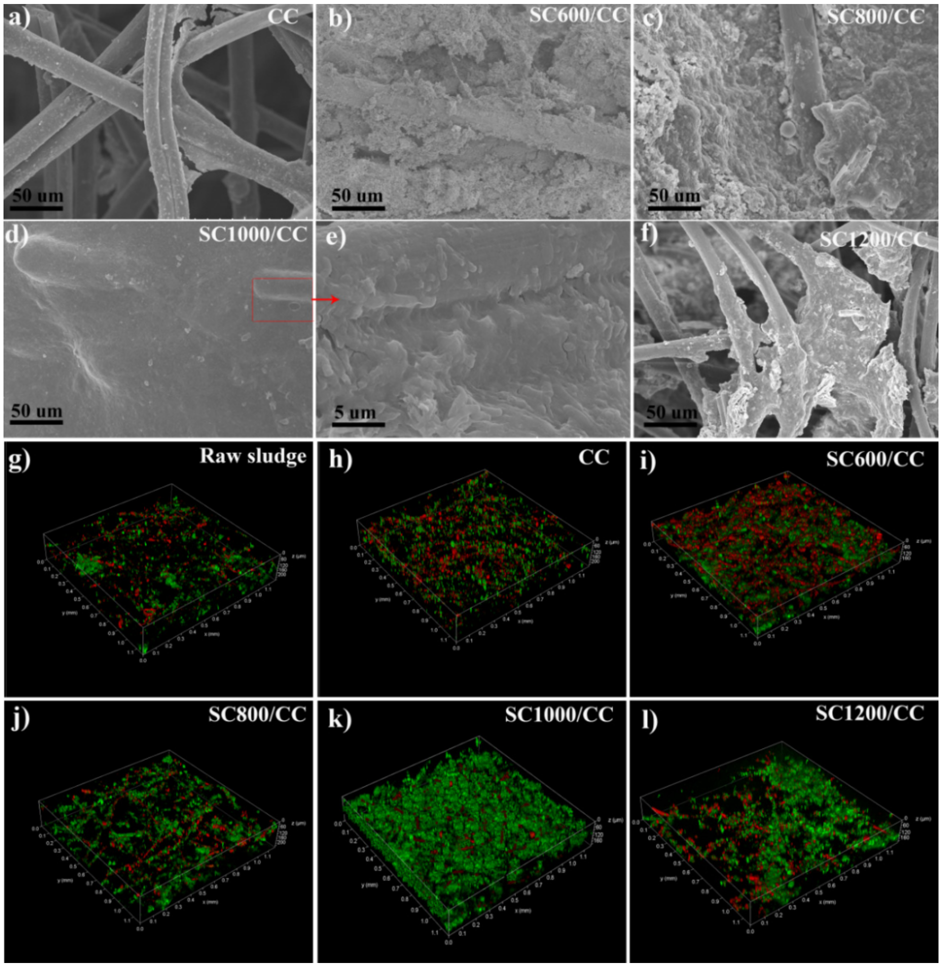

Figure S8. SEM and CLSM images of raw sludge and anodes biofilms from MFCs after 54 d of batch mode operations. e) The amplified SEM images of Fig. 6d. Metabolically active cells are green and inactive cells are red

The SC1000/CC formed a dense electroactive biofilm, the interface interaction increased. At the anode, rod-shaped bacteria were densely embedded in the extracellular polymer and were in close contact with the electrode, and the EPS-encapsulated microbial community had a stable three-dimensional structure, forming a safe and closely connected microenvironment, which is conducive to the transmission of electrons. It is speculated that it is related to the electroactive bacterium Geobacter [5, 7]. In the thick biofilm, Geobacter can actively secrete c-

type cytochrome oxidoreductase, assist its extracellular respiration and promote extracellular electron transfer [2, 3, 5].

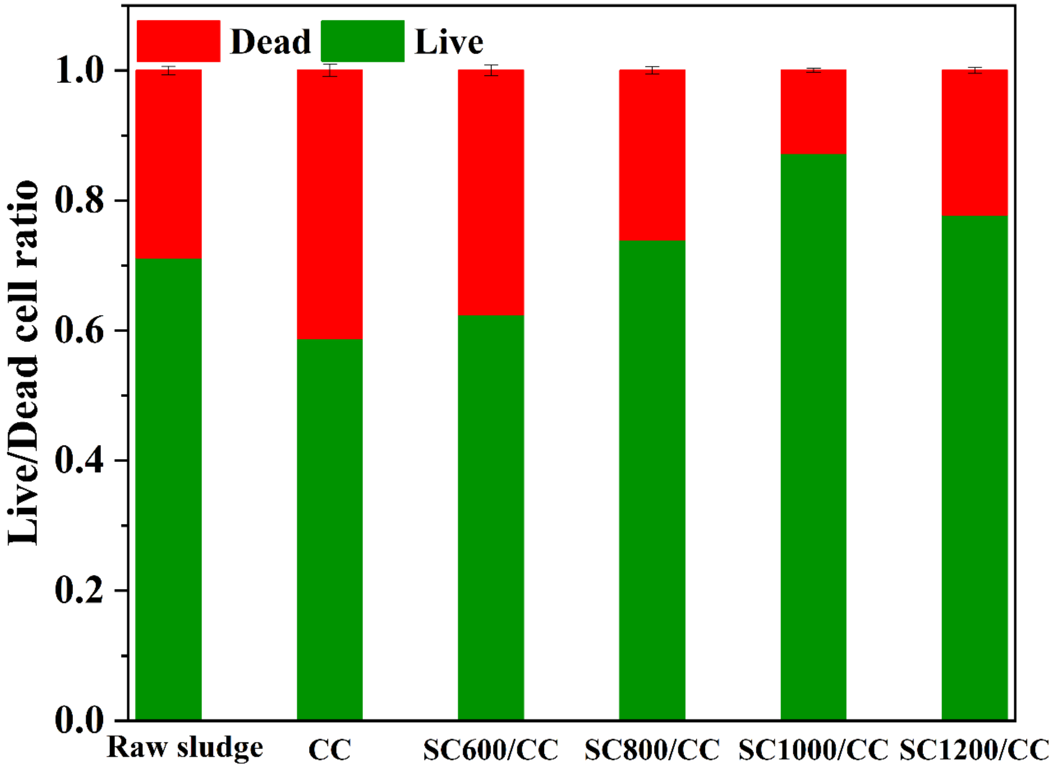

Figure S9. Ratio of dead and alive cells for raw inoculated sludge and anode biofilms at MFCs.

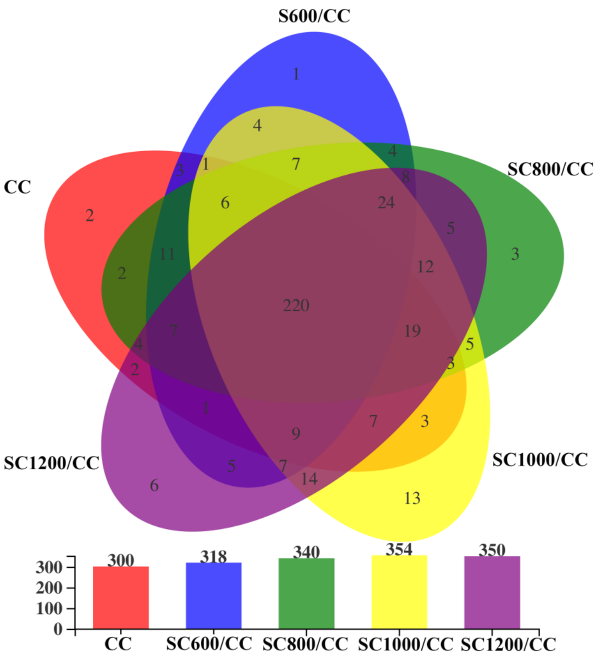

Figure S10. Venn diagram of the biofilms attached on CC and SC anodes.

The diversity of the bacterial community can ensure the stability of anode biofilms, effective decomposition of organic matter in the anode chamber and improve the consumption of COD and the production of bioenergy. The Shannon index (3.531) and the total number of species (354) of SC1000/CC were the largest among all the anodes (Figure S10), indicating that the anode has the most abundant microbial diversity, which is also the reason for the denser biofilm on the surface of the SC1000/CC electrode (Figure S8e). Interestingly, the Simpson (0.114) and Berger-Parker index (0.305) of SC1000/CC were also the largest among all anodes, indicating that the anode has obvious dominant flora and selective enrichment of functional microorganisms

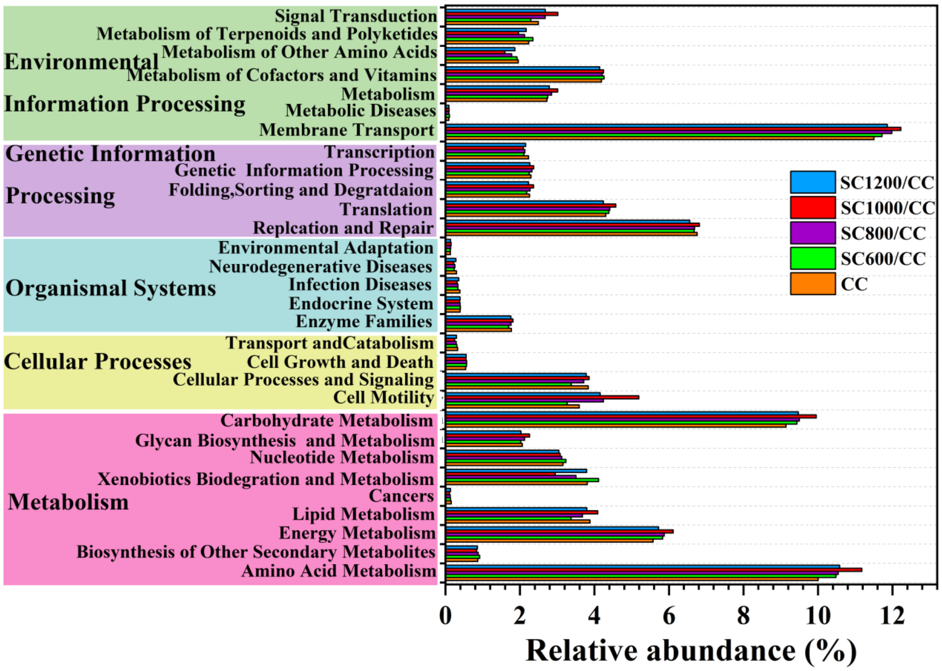

Figure S11. Predictive abundances of metagenomic functional genes on the anode biofilms based on PICRUSt of KEGG analysis.

PTCRUST function prediction explained the metabolic pathways and differences in microorganisms in the anode biofilms (Figure S11). Cell movement is the main pathway in clusters for cellular processes. In the SC1000/CC anode biofilm, the relative abundance of cell mobility was the highest, indicating that the cell activity on the biofilm was the highest, which is consistent with the results in Figure S8 and Figure S9. In the genetic information processing cluster, replication, repair, and translation were the main metabolic pathways. Enzymes

accounted for the largest proportion of the organic system cluster. In the environmental information processing cluster, membrane transport, signal transduction, and metabolism of cofactors and vitamins were the main metabolic pathways. The SC1000/CC anode biofilm accounted for the largest proportion, which is the reason that the electroactive biofilm had high electrocatalysis.

Table S1. PBS solution composition

Composition	Concentration（$g \cdot L^{-1}$）
NH_4Cl	0.31
KCl	0.13
$NaH_2PO_4 \cdot 2H_2O$	3.32
$Na_2HPO_4 \cdot 12H_2O$	10.32

Table S2. Trace element solution composition

Composition	Concentration（$g \cdot L^{-1}$）
$C_6H_9NO_6$	2.0
$MgSO_4 \cdot 7H_2O$	3.0
$MnSO_4 \cdot H_2O$	0.5
NaCl	1.0
$FeSO_4 \cdot 7H_2O$	0.1
$CaCl_2 \cdot 2H_2O$	0.1
$CoCl_2 \cdot 6H_2O$	0.1
$ZnSO_4 \cdot 7H_2O$	0.13
$CuSO_4 \cdot 5H_2O$	0.01
$AlK(SO_4)_2 \cdot 12H_2O$	0.01
H_3BO_3	0.01
$Na_2MoO_4 \cdot 2H_2O$	0.025
$NiCl_2 \cdot 6H_2O$	0.024
$Na_2WO_4 \cdot 2H_2O$	0.025

Table S3. Vitamin solution

Composition	Concentration（$g \cdot L^{-1}$）
Biotin	2.0
Folic acid	2.0
Pyridoxine	10.0
Thiam	5.0
Nicotinic acid	5.0
Cobalamin	0.1
P-aminobenzoic acid	5.0
Thioctic acid	5.0

Table S4. Specific area and total surface area of SC samples

Samples	BET ($m^2\ g^{-1}$)	Total pore volume ($cm^{-3}\ g^{-1}$)	Micropore volume ($cm^{-3}\ g^{-1}$)	Average pore width (nm)
SC600	96.12	0.1824	0.0179	8.3071
SC800	156.04	0.2108	0.0259	8.8716
SC1000	216.00	0.2055	0.0367	8.7108
SC1200	176.55	0.2033	0.0268	8.5818

Table S5. Atomic contents of SC samples

Element (%)	SC600	SC800	SC1000	SC1200
C	32.05	37.69	57.03	63.98
O	41.96	38.29	25.08	23.17
N	3.32	2.12	1.88	1.71
P	1.74	1.83	1.86	--
S	0.03	--	0.18	0.25
Si	8.93	8.66	5.24	4.91
K	0.36	0.29	0.22	0.15
Ca	1.20	1.06	1.00	0.64
Mg	0.67	0.45	0.47	0.47
Al	1.29	1.15	1.00	0.70
Fe	7.56	7.43	5.49	3.78
Cu	0.34	0.32	0.30	0.18
Zn	0.28	0.30	0.25	--
Ti	0.27	0.13	0.20	--

Table S6. Species diversity and abundance indexes

Samples	Shannon	Simpson	Ace	Chao	Berger-Parker
CC	3.218	0.093	246.38	258.05	0.229
SC600/CC	3.271	0.087	250.84	254.33	0.216
SC800/CC	3.305	0.063	259.79	258.44	0.158
SC1000/CC	3.531	0.114	257.44	261.43	0.305
SC1200/CC	3.520	0.074	253.79	263.38	0.190

References

[1] A.P. Borole.; G. Reguera.; B. Ringeisen.; Z.W. Wang.; Y. Feng.; B.H. Kim. Electroactive biofilms: Current status and future research needs. Energy & Environmental Science. **2011**, *4*, 4813-4834.

[2] Y. Gu.; V. Srikanth.; A.I. Salazar-Morales.; R. Jain.; J.P. O'Brien.; S.M. Yi.; R.K. Soni.; F.A. Samatey.; S.E. Yalcin.; N.S. Malvankar. Structure of Geobacter pili reveals secretory rather than nanowire behaviour. Nature. **2021**, *597*, 430-434.

[3] F.B. Wang.; Y.Q. Gu.; J.P. O'Brien.; S.M. Yi.; S.E. Yalci.; V. Srikanth.; C. Shen.; D. Vu.; N.L. Ing.; A.I. Hochbaum.; E.H. Egelman.; N.S. Malvankar. Structure of Microbial Nanowires Reveals Stacked Hemes that Transport Electrons over Micrometers. Cell. **2019**, *177*, 361-362.

[4] D.R. Lovley. Bug juice: harvesting electricity with microorganisms. Nat Rev Microbiol. **2006**, *4*, 497-508.

[5] D.R. Lovley. Electromicrobiology. Annu Rev Microbiol. **2012**, *66*, 391-409.

[6] C.I. Torres.; A.K. Marcus.; H.S. Lee.; P. Parameswaran.; R. Krajmalnik-Brown.; B.E. Rittmann. A kinetic perspective on extracellular electron transfer by anode-respiring bacteria. FEMS Microbiol Rev. **2010**, *34*, 3-17.

[7] B.E. Logan.; R. Rossi.; A. Ragab.; P.E. Saikaly. Electroactive microorganisms in bioelectrochemical systems. Nat Rev Microbiol. **2019**, *17*, 307-319.
